# Twentieth century occurrence of the Long-Beaked Echidna *Zaglossus bruijnii* in the Kimberley region of Australia

**DOI:** 10.3897/zookeys.255.3774

**Published:** 2012-12-28

**Authors:** Kristofer M. Helgen, Roberto Portela Miguez, James L. Kohen, Lauren E. Helgen

**Affiliations:** 1 Division of Mammals, Department of Vertebrate Zoology, National Museum of Natural History, Smithsonian Institution, P.O. Box 37012, MRC 108, Washington, D.C. 20013-7012, USA; 2 Mammal Section, Department of Life Sciences, The Natural History Museum, Cromwell Road, London SW7 5BD, UK; 3 Department of Biological Sciences, Macquarie University, North Ryde, NSW 2109, Australia

**Keywords:** Extinction, Kimberley, monotreme, Pleistocene survival, rock art, *Zaglossus*

## Abstract

The monotreme genus *Zaglossus*, the largest egg-laying mammal, comprises several endangered taxa today known only from New Guinea. *Zaglossus* is considered to be extinct in Australia, where its apparent occurrence (in addition to the large echidna genus *Megalibgwilia*) is recorded by Pleistocene fossil remains, as well as from convincing representations in Aboriginal rock art from Arnhem Land (Northern Territory). Here we report on the existence and history of a well documented but previously overlooked museum specimen (skin and skull) of the Western Long-Beaked Echidna (*Zaglossus bruijnii*) collected by John T. Tunney at Mount Anderson in the West Kimberley region of northern Western Australia in 1901, now deposited in the Natural History Museum, London. Possible accounts from living memory of *Zaglossus* are provided by Aboriginal inhabitants from Kununurra in the East Kimberley. We conclude that, like *Tachyglossus*, *Zaglossus* is part of the modern fauna of the Kimberley region of Western Australia, where it apparently survived as a rare element into the twentieth century, and may still survive.

## Introduction

**Figure 1.**
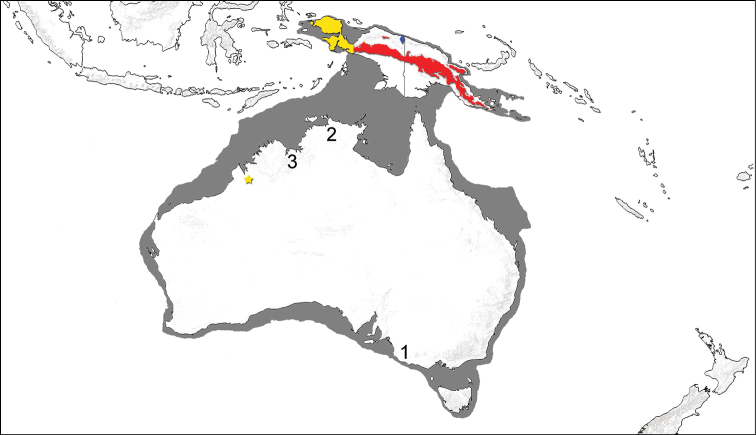
Map of the Greater Australian continent. Map includes an outline of the larger land mass know as “Sahul” or “Meganesia” that forms when the continental shelf (dark grey) is exposed during glaciation. Overlaid is the modern distribution of the three recognized species of *Zaglossus*: *Zaglossus bartoni* (red), *Zaglossus bruijnii* (yellow), and *Zaglossus attenboroughi* (blue diamond), with the Kimberley record of *Zaglossus bruijnii* highlighted by a yellow star. Other possible Australian records of *Zaglossus* cf. *bruijnii* are numbered by general locality: **1** Pleistocene fossil remains from Naracoorte, South Australia, referred to *Zaglossus* cf. *bruijnii* by Murray (1978a) **2** Aboriginal rock art (probably late Pleistocene in age) from Arnhem Land, Northern Territory (see Figure 2) **3** Aboriginal reports of a second, larger echidna taxon, in addition to *Tachyglossus*, present in the East Kimberley (Kununurra, Western Australia) in recent (20th century) memory (see text).

The egg-laying mammals, or monotremes (Monotremata), are the sister group to all other extant mammals and are known as living animals only from the Australian continent, incorporating the modern landmasses of Tasmania, Australia, and New Guinea, which share a continental shelf that is periodically united during times of lowered sea levels as a single continuous landmass (“Sahul” or “Meganesia”). There are two extant monotreme families. The platypus, Ornithorhynchidae, is represented by a single living genus and species, *Ornithorhynchus anatinus* (Shaw, 1799), a semi-aquatic monotreme distributed throughout eastern Australia from tropical Queensland south to Tasmania and Kangaroo Island. The echidnas, Tachyglossidae, are classified in two living genera, the smaller short-beaked echidna (genus *Tachyglossus*), represented by one species, *Tachyglossus aculeatus* (Shaw, 1792), and the larger long-beaked echidnas (genus *Zaglossus*), with three living species currently recognized (Flannery and Groves 1998). *Tachyglossus aculeatus* is one of the most widely distributed Meganesian mammals, occurring in a wide range of habitats throughout Tasmania, Australia, and much of New Guinea. The long-beaked echidnas, today known only from New Guinea, are inhabitants of rainforests and subalpine meadows—*Zaglossus bruijnii* (Peters and Doria, 1876), distributed in western New Guinea, *Zaglossus bartoni* (Thomas, 1907a), distributed in central and eastern New Guinea, mainly at higher elevations, and *Zaglossus attenboroughi* Flannery and Groves, 1998, reported to date only from the Cyclops Mountains (Flannery and Groves 1998, Groves 2005, Baillie et al. 2009), an outlying mountain range along the north coast of New Guinea (Figure 1).

**Figure 2.**
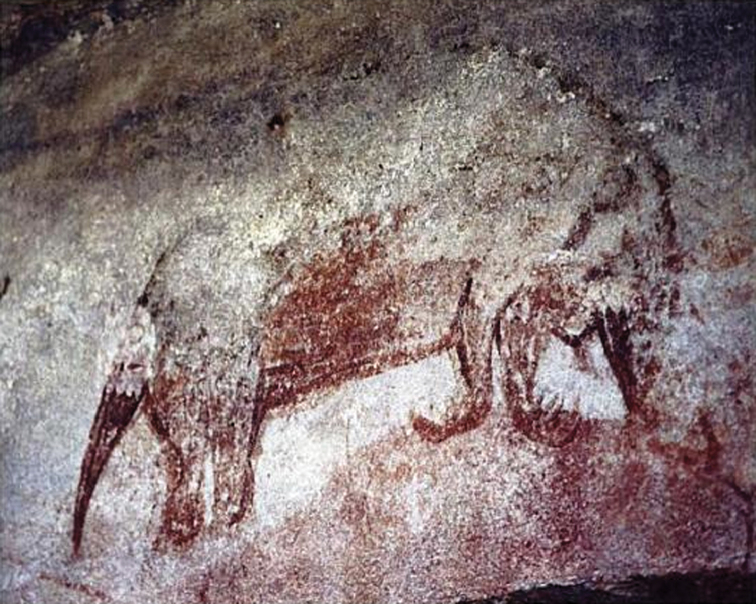
Australian rock art of *Zaglossus*. Photograph of an Aboriginal rock art illustration from Arnhem Land depicting the characteristic long and down-curved beak (and whitish head of some specimens) of *Zaglossus* (see Murray and Chaloupka 1984). Photograph by G. Chaloupka.

Though *Tachyglossus* is regarded as the only extant echidna in Australia, until the late Pleistocene several additional tachyglossids, all larger than *Tachyglossus*, occurred in Australia. *Megalibgwilia owenii* (Krefft, 1868) (often called *Megalibgwilia ramsayi*, a junior synonym, in current literature) was a *Zaglossus*-sized echidna (estimated mass *circa* 10 kg, but more robust than *Zaglossus* and with a less elongate or downcurved rostrum) known from Pleistocene localities in New South Wales (Wellington Caves), South Australia (Naracoorte), Tasmania (Montagu Caves and King Island), and south-western Western Australia (Tight Entrance Cave) (Murray 1978b, Griffiths et al. 1991, Turney et al. 2008, Prideaux et al. 2010), indicating a distribution centered on more temperate regions of the continent. “*Zaglossus*” *hacketti* Glauert, 1914, the largest monotreme yet discovered (estimated mass *circa* 20 kg), is documented only from Pleistocene postcranial remains from south-western Western Australia (Mammoth Cave); its generic placement has always been provisional pending the discovery of cranial material or detailed comparative taxonomic study of tachyglossid postcrania (Griffiths et al. 1991, Long et al. 2002). Postcranial remains of a relatively gracile *Zaglossus*-sized echidna, provisionally referred to the living *Zaglossus bruijnii*, have been reported from the Pleistocene of South Australia (Henschke’s Quarry Cave at Naracoorte) (Murray 1978a [but see Pledge (1980), who considered this more likely a “giant *Tachyglossus*”]), and Aboriginal rock art corresponding to *Zaglossus* (*Zaglossus* cf. *bruijnii*) is compellingly recorded from Arnhem Land, Northern Territory (Murray and Chaloupka 1984, Lewis 1986, Chaloupka and Murray 1986) (Figure 2). Thus, at least four echidna species, *Tachyglossus aculeatus*, *Megalibgwilia oweni*, *“Z.” hacketti*, and *Zaglossus* cf. *bruijnii*, constitute the known Quaternary tachyglossid fauna of the Australian continent south of New Guinea.

**Figure 3.**
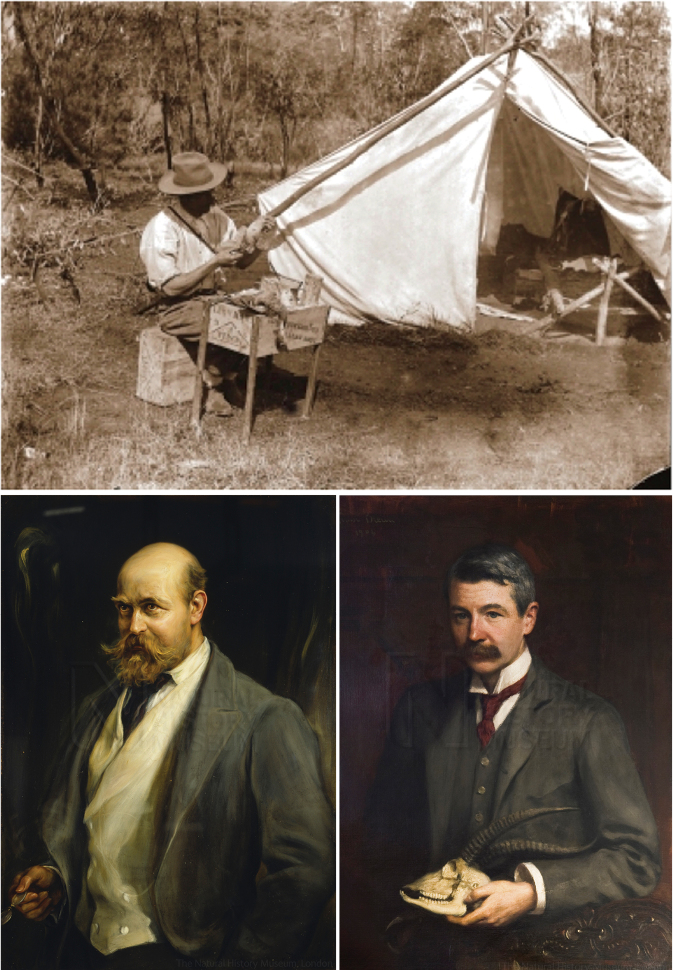
*Dramatis personae*. Clockwise from top: Australian natural history collector John T. Tunney (1871–1929), preparing specimens on the northern Australian expeditionary efforts during which his *Zaglossus* specimen was collected; M.R. (Michael Rogers) Oldfield Thomas (1858–1929), mammal taxonomist at the British Museum (Natural History), London, who studied the Tunney *Zaglossus* specimen; Lord L. (Lionel) Walter Rothschild (1868–1937), eccentric collector and naturalist who used his family fortune to amass a very large personal scientific collection, which became the Zoological Museum at Tring and included the Tunney *Zaglossus* specimen. Tunney portrait courtesy of the Western Australian Museum, Perth; Rothschild and Thomas portraits courtesy of the Natural History Museum, London.

Here we report an overlooked modern museum specimen (skin, skull, and forelimb skeleton) of *Zaglossus* that was apparently collected in 1901 in the West Kimberley region of north-western Australia by the Australian naturalist and collector John T. Tunney (Figure 3). Based on an agreement between Lord L. Walter Rothschild, the eccentric naturalist who built up an astonishingly large personal collection of natural history specimens in his private museum in Tring (in the county Hertfordshire outside of London), and Bernard Henry Woodward, the London-born director of the Western Australian Museum in Perth, Tunney was commissioned by Rothschild to travel through some of the most remote areas of northern Australia in the first years of the twentieth century in order to collect butterflies, moths, mammals, and birds for Tring, and Aboriginal cultural artifacts for the museum at Perth. From April 1901 to November 1903, in a pioneering effort, Tunney collected natural history specimens and cultural artifacts along a transect that extended from the Pilbara Region in Western Australia to the South Alligator River in Northern Territory, before returning to Perth (Thomas 1904a, Hartert 1905, Whittell 1954, Storr 1966, Gray 2003, Chadwick 2008). On this northern Australian expedition, Tunney obtained the first specimens of various mammals previously unknown to science, including the small dasyurid marsupial *Antechinus bellus* (Thomas, 1904a), the rats *Rattus tunneyi* (Thomas, 1904a) and *Rattus colletti* (Thomas, 1904c), and two larger mammals, both kangaroos—the Black Wallaroo *Macropus bernardus* (Rothschild 1904) and Rothschild’s Rock-Wallaby *Petrogale rothschildi* (Thomas, 1904b). Tunney also collected what were to become the type specimens of several other then-undescribed mammal taxa known by a few other museum specimens at the time, including the small dasyurid marsupial *Phascogale pirata* Thomas, 1904a; the West Kimberley and Northern Territory subspecies of the wallaroo (*Macropus robustus woodwardi* Thomas, 1901, and *Macropus robustus alligatoris* Thomas, 1904a, respectively); and the Arnhem Land subspecies of the Nabarlek or Pygmy Rock-Wallaby, *Petrogale concinna canescens* Thomas, 1909.

Despite the importance of Tunney’s mammalogical collections, no full report on these materials has ever been published. The most important account is M.R. Oldfield Thomas’ (1904a) preliminary discussion written after receipt and early review of Tunney’s material received at the Tring Museum. (The Tunney collection was transferred, along with the rest of Rothschild’s mammal collections, from Tring to the Natural History Museum, London, in 1939 following Rothschild’s death in 1937.) One of the most important Tunney specimens that was never critically reported is what appears to be a north-western Australian specimen of *Zaglossus* collected in 1901, which we discuss here. This specimenchallenges current thinking about the timing of extinction of the genus in Australia and offers new insight into northern Australian biogeography and the ecology of this critically endangered monotreme lineage.

## Methods

### Museums

Specimens discussed in this paper are stored in the collections of the American Museum of Natural History, New York , USA (AMNH); the Natural History Museum, London, UK (BMNH); the Museum of Comparative Zoology, Harvard University, Cambridge, Massachusetts, USA (MCZ); the Museum Zoologicum Bogor, Cibinong, Indonesia (MZB); the National Museum of Natural History, Smithsonian Institution, Washington, D.C., USA (USNM); and the Western Australian Museum, Perth, Australia (WAM).

## Results

### The Kimberley Zaglossus specimen

**Figure 4.**
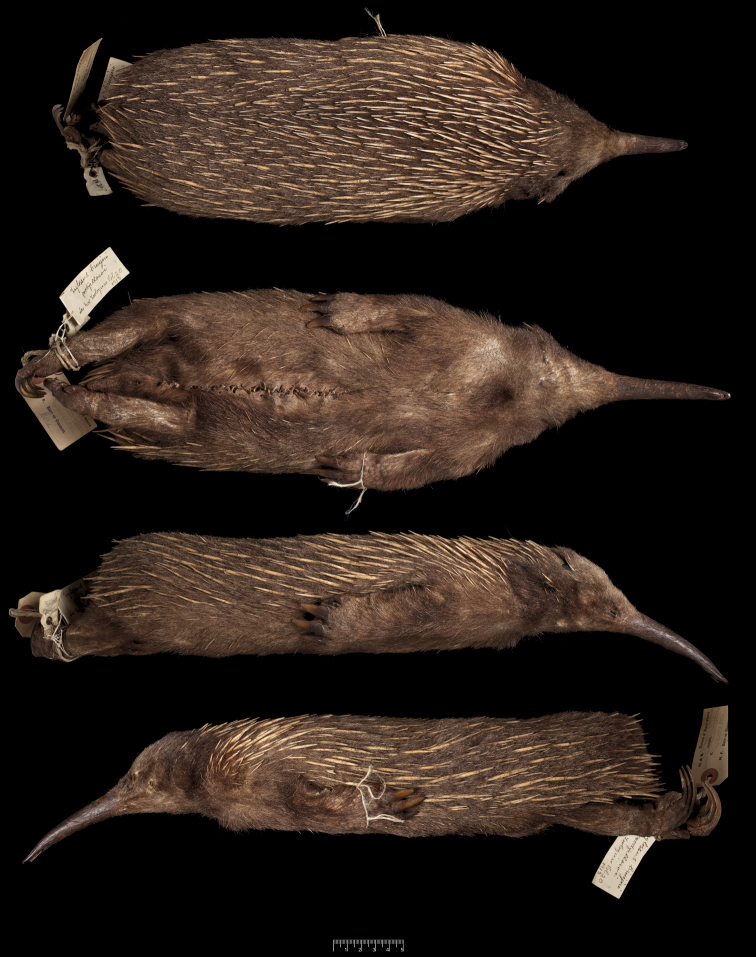
Study skin of the Kimberley *Zaglossus* (BMNH 1939.3315), bearing the original field tags of John T. Tunney. From top: dorsal, ventral, right lateral, and left lateral views. Scale bar = 5 cm.

**Figure 5.**
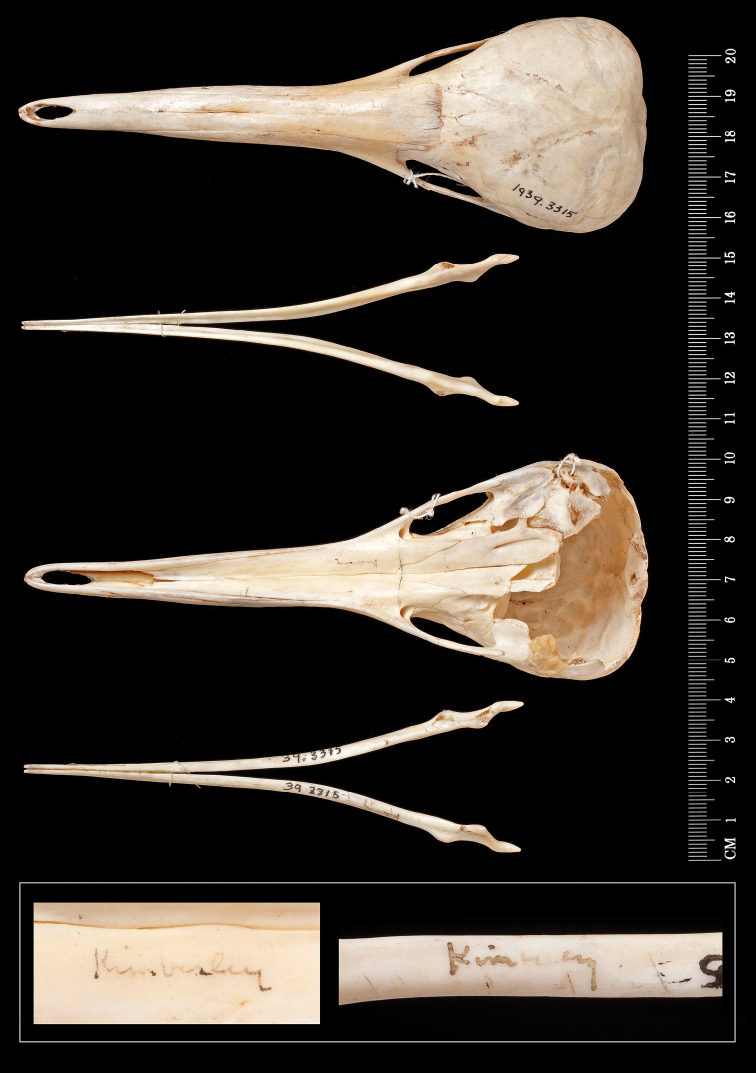
Cranium and dentaries of the Kimberley *Zaglossus* (BMNH 1939.3315). From top: dorsal view of the cranium, dorsal view of the dentaries, ventral view of the cranium, ventral view of the dentaries, and, at bottom, close-up views of Thomas’ labeling of “Kimberley” on the specimen’s palate (left) and dentary (right). Scale bar = 20 cm.

**Figure 6.**
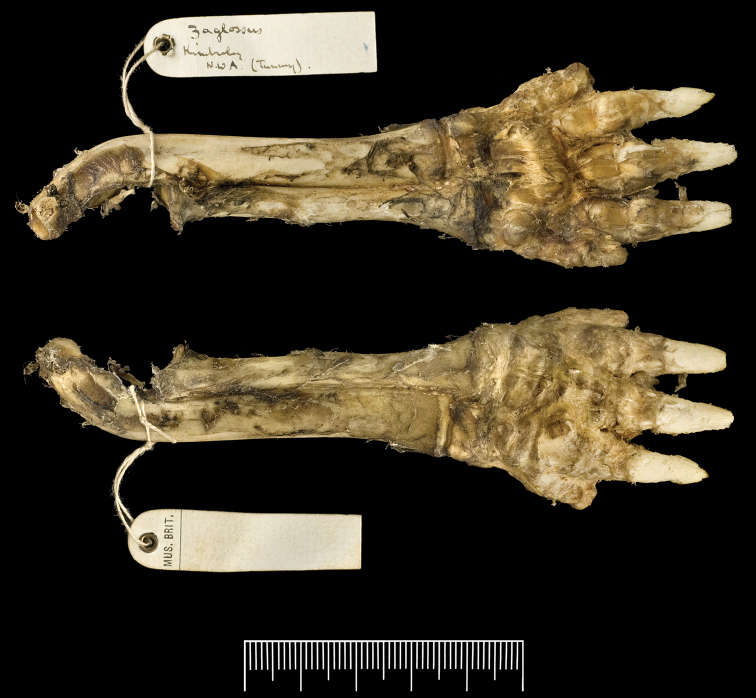
Articulated right forelimb elements of the Kimberley *Zaglossus* (BMNH 1939.3315). Associated label notes “Zaglossus Kimberley N.W.A. (Tunney)” in Thomas’ handwriting. Ventral view above, dorsal view below. Scale bar = 5 cm.

**Figure 7.**
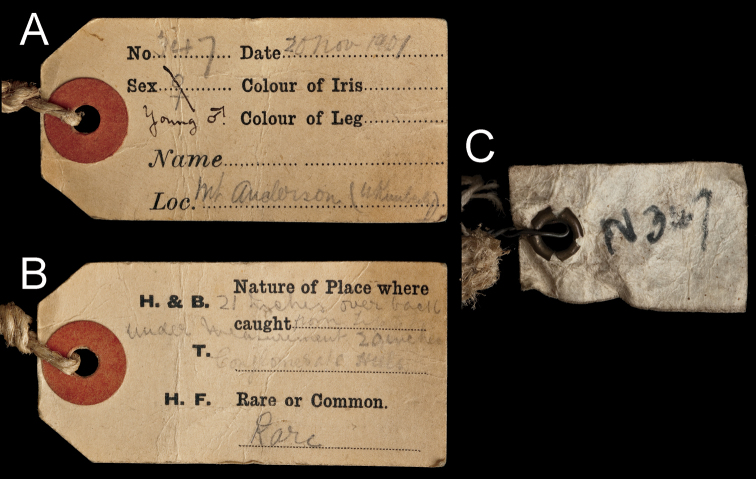
John Tunney’s original field labels attached to the skin of BMNH 1939.3315. **A** front of card skin tag (attached with sturdy twine to right ankle of study skin) bearing original data, providing the specimen’s field number, date of collection, age and sex, and locality **B** back of same card skin tag bearing original data, detailing the specimen’s measurements, context of collection, and abundance **C** cloth tag bearing original field number (“N 347”), wired tightly to right ankle of study skin. See text for details.

**Figure 8.**
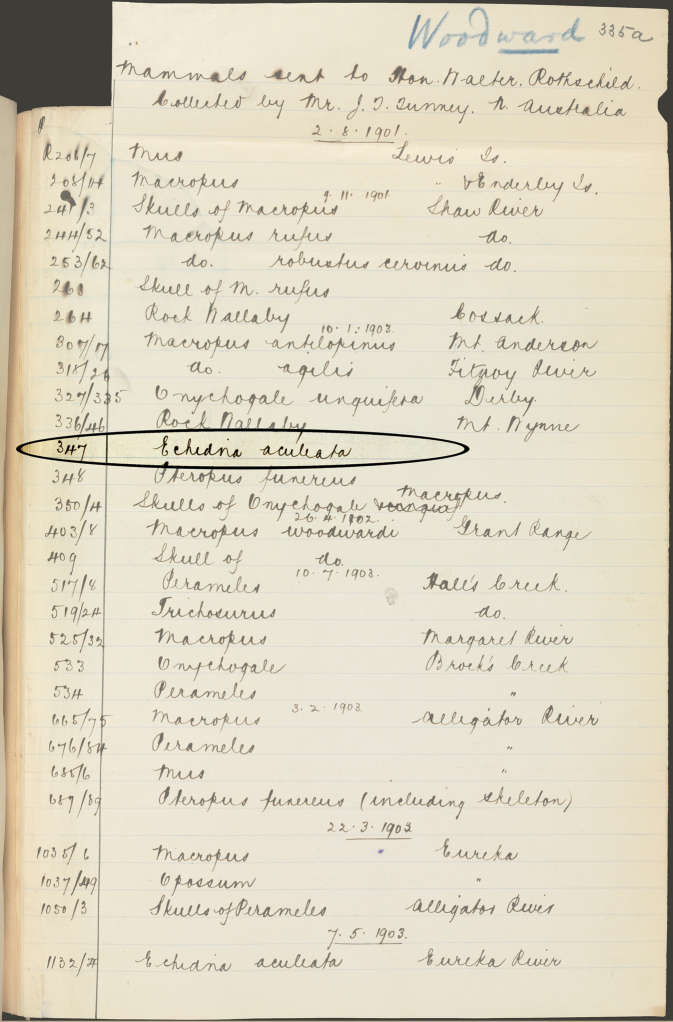
Specimen export list. A list of specimens shipped from Perth to Tring included in a letter, dated 25 April 1904, from Bernard Woodward at the Western Australian Museum to Oldfield Thomas, detailing the transfer of Tunney specimens to Rothschild at Tring. The list includes his number 347 (now BMNH 1939.3315), an echidna identified as “*Echidna aculeata*” (i.e. *Tachyglossus aculeatus*) prior to Thomas’ examination of the specimen in London, where he realized it is a *Zaglossus*; we have circled and highlighted this entry in the list.

**Figure 9.**
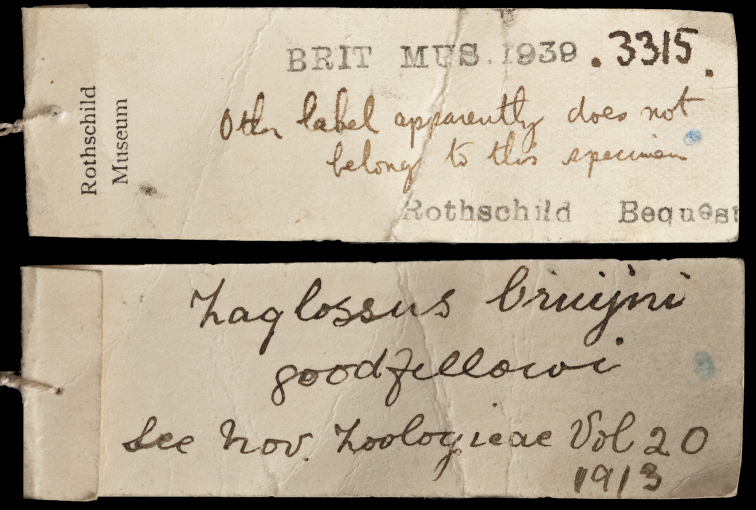
Non-original tag (views of front and back) added at Tring or BMNH, and apparently bearing the handwriting of Tring taxidermist Fred Young. The tag bears a note suggesting that the original labels must be incorrect because the specimen corresponds to Rothschild’s concept of *Zaglossus bruijni goodfellowi*, then considered endemic to the Indonesian island of Salawati, following his 1913 key (Rothschild 1913). The note seems to indicate that knowledge of the importance of this specimen has been obscured since or before the specimen’s transfer from Tring to BMNH, as it was assumed by Young to be a mistake.

During a visit to the Natural History Museum, London, in 2009, the first author studied a museum skin of *Zaglossus bruijnii* (BMNH 1939.3315; Figures 4–9), bearing original tags from John T. Tunney, stored among supposedly unprovenanced specimens of *Zaglossus*. This skin also has an associated cranium, mandibles, and distal right forelimb elements, which were extracted from the study skin early in the twentieth century (see below). The tags record the collection of this specimen from Mount Anderson, an inland locality in the West Kimberley region of north-western Western Australia, on 20 November 1901 (Figure 7).

Tunney’s typical tags (used by Tunney and some other collectors from the Western Australian Museum in Perth) were strong card tags preprinted with the following categories (see figure in Chadwick [2008]): (on one side of the card) “No.” (i.e., field number), “Date” (i.e., date of collection), “Sex” (i.e., male or female), “Colour of Iris” (i.e., eye color, used by Tunney for birds, but generally not for mammals), “Colour of Leg” (used by Tunney for birds, but not for mammals), “Name” (given by Tunney either as the scientific or common name of the species), “Loc.” (i.e., the collection locality); and (on the other side of the card) “Nature of Place where caught” (i.e., habitat), “Rare or Common” (i.e., an indication of abundance), “H + B.” (i.e., length of head and body), “T.” (i.e., length of tail), and “H. F.” (i.e., length of hind foot).

Tunney’s tag, written in his characteristic handwriting, and tied with thick sturdy string to the right hindfoot of the specimen (Figures 4 and 7), bears an original field number (347), a date (“20 Nov. 1901”), reports the specimen’s collection from “conglomerate hills” (“Nature of Place where caught”) at “Mt Anderson (W Kimberley)” (locality), and indicates that it was “Rare” (a classification only occasionally reported on his mammal tags). Tunney originally marked the sex of the animal as female (“♀”), which was later corrected in pen on the tag to “young ♂” (reflecting a mammal difficult to sex, as echidnas can be). Tunney left the “Name” field blank on his tag, which is somewhat unusual—he usually reported a scientific or common name on his mammal tags. This may indicate that Tunney was uncertain exactly what species he had before him. Tunney also usually reported standard length measurements on tags for his mammal and bird specimens (i.e., head-body, tail, and hind foot lengths), but in this case he gave the measurements of the specimen only as “21 inches over back from tip to tip” and “under measurement 20 inches”, indicating a mammal for which the head-body and tail lengths were unusually difficult to measure. Thetotal study skin, as now prepared, still measures about 21 inches measured over the dorsum and 20 inches measured along the underside. The specimen also bears a smaller field tag, worn and dirty, that is made of cloth-like paper, attached to the right hindleg with wire, and bearing only the field number, “N 347” (Figure 7).

The specimen is a well-made study skin, with the hindlegs directed posteriorly and the forelegs folded back against the underside (Figure 4). It was originally prepared with the skull and parts of the limbs retained intact inside the skin (the skull and right forelimb were apparently later removed from the skin and prepared in England—see below). The pelage is quite pale brown, and the specimen is rather sparsely furred, with mostly white spines, and has spines invading the sides of the belly, claws only on the middle three digits of both forefeet and both hindfeet, and hindleg spurs.

This specimen was misidentified as a Short-beaked echidna before its skull was extracted and studied. Identified by field number (347), it was listed as “*Echidna aculeata*” (i.e., *Tachyglossus aculeatus*) when it was sent from Australia to Rothschild at Tring, and identified as such in a letter dated 25 April 1904 sent by Bernard Woodward to Oldfield Thomas in London, discussing details of the mammal specimens collected by Tunney (Figure 8). Soon after its arrival at Tring, ectoparasite specimens taken from this skin formed the basis for the description of a new species of tick, *Amblyomma australiense*, by Neumann (1905), and a new species of flea, *Echidnophaga liopus*, by Jordan and Rothschild (1906). Both of these publications still listed the identity of the host as “*Echidna aculeata*” (these parasitological discoveries are discussed further below).

Though the identification of this specimen as *Zaglossus* has gone unreported in the literature until now, we are not the first researchers to notice that this specimen provides a modern record for *Zaglossus* from Western Australia. Oldfield Thomas, arguably the greatest mammalogical taxonomist of all time, examined Tunney’s specimens when they arrived in England, and made notes that indicate he understood Tunney’s specimen was a Kimberley *Zaglossus*. Thomas would have known that the Tunney skin in question was a *Zaglossus* rather than a *Tachyglossus* the moment he saw it, even if Rothschild was unaware of this. Thomas apparently removed the skull (the skull, by its lack of sutural ossifications, shows the animal to be a nearly mature subadult) and the bones of the right forelimb (articulated radius, ulna, and forefoot) from the study skin. The skull is intact apart from some missing basicranial fragments and is labelled “Kimberley” in Thomas’ handwriting on the palate (Figure 5); it also bears two labels in Thomas’ handwriting, one identifying the specimen as an “imm[ature]. Zaglossus, coll[ected by]. Tunney” and the other noting that the skull compares favorably to an immature specimen of *Zaglossus bruijnii* from Fakfak (western New Guinea) preserved in the Zoological Museum in Amsterdam. The dentary is also marked in ink with the word “Kimberley” in Thomas’ handwriting (Figure 5). Thomas labeled the forelimb “Zaglossus Kimberley N.W.A. (Tunney)” (i.e., N.W.A. = north-western Australia) (Figure 6). These labels indicate to us that Thomas recognized that the specimen was indeed a *Zaglossus*, and that he was satisfied that it had been collected by Tunney in the Kimberley region of Australia. We suspect that Thomas extracted the right forelimb elements from the skin of the specimen to see if its humerus was preserved. He would have wanted to compare it to the humeri of the large fossil echidnas that had previously been described from Australia; the holotypes of two large echidna taxa described from the Australian Pleistocene (*Echidna owenii* Krefft, 1868, *Echidna ramsayi* Owen, 1884, now classified in the genus *Megalibgwilia*) are right humeri (Mahoney and Ride 1975). Only the radius, ulna, and distal elements of the manus were present in the skin, however. An x-ray of the skin confirms that the right forelimb and skull of this specimen were indeed extracted from the skin, with their impressions, still evident inside the skin, closely matching the osteological elements.

It is not clear on what date Thomas extracted the skull and forelimb of the Tunney specimen, but he may have written the accompanying labels after 1907 (or replaced them with newer labels if he had written them earlier), because until at least 1907 Thomas was apparently under the impression that *Acanthoglossus* (rather than *Zaglossus*) was the correct generic name for the long-beaked echidnas (Thomas 1907a, 1907b), though usage of this name wavered at the time. Universally accepted usage of *Zaglossus* Gill 1877, which predates the generic synonyms *Acanthoglossus* Gervais 1877a and *Proechidna* Gervais 1877b(names which had previously enjoyed wide usage), followed from Allen’s (1912) influential monograph on the genus, though earlier authors, including Rothschild, had regularly pointed out that *Zaglossus* was the earliest generic name (e.g., Gill 1885, Palmer 1895, Coues 1895, Rothschild 1905a). In any case, Thomas died in 1929 (Thomas and Tunney both passed away in June 1929) without discussing Tunney’s *Zaglossus* specimen in any publication, which is surprising considering that Thomas was such a prolific author of papers on mammals (he produced approximately 1100 publications on mammal taxonomy, naming 2900 mammal taxa [Hill 1990]). We can identify several reasons why Thomas may not have prioritized publishing a note about this specimen. The most important might be Rothschild’s eccentric penchant for echidnas (along with kangaroos—similar to his personal interest in ratites amongst his ornithological collections [M. Rothschild 1983]), which may have excluded Thomas from freely publishing on these holdings at Tring. Rothschild permitted Thomas to publish many papers based on Tring mammals, but Rothschild was very fond of kangaroos and echidnas, keeping various kinds alive at his family’s estate (M. Rothschild 1983), and it seems he preferred to publish reviews of Tring’s kangaroos and echidnas on his own, except perhaps where Thomas chose to name them in Rothschild’s honor (as in the case of *Petrogale rothschildi* Thomas, 1904b). This may have suited Thomas fine, as Rothschild’s publications on kangaroos and echidnas were often premature and incompletely prepared and reasoned (e.g., Rothschild 1903, 1904, 1905b, 1905c, 1907) (with some important exceptions, such as the tree kangaroo monograph published by Rothschild and Dollman 1936), in general falling far short of Thomas’ authoritative command of these groups, which was established early in Thomas’ career (Thomas 1888). Another reason that Thomas did not publish on the specimen could be that it was not clearly describable as a new taxon (the Thomasian special focus), the single specimen available being indistinguishable from specimens of *Zaglossus bruijnii* from western New Guinea. With the wealth of clearly new mammal taxa Thomas had available to describe, he may have set this echidna specimen to the side, hoping that additional Australian specimens, especially a mature specimen or a series, might become available so that he could better understand the characteristics of the Kimberley *Zaglossus*.

While Thomas’ impressions as to the identity of the Tunney *Zaglossus* specimen seem clear, it is not clear whether Rothschild was aware that the specimen was a *Zaglossus*,or if so, whether he accepted its authenticity. Rothschild published several observations on echidna taxonomy (Rothschild 1892, 1905a, 1913), including one co-authored with Thomas (Thomas and Rothschild 1922), and one in which Thomas abstained or was excluded from authorship (Rothschild, in Thomas and Rothschild 1922). Like Thomas, Rothschild never mentioned Tunney’s *Zaglossus* specimen in a publication before he died in 1937. In 1939, the Tring mammal collection was transferred to the BMNH, and most of it is now housed at South Kensington. This is when the echidna specimen was given the BMNH accession number 1939.3315. The BMNH Mammalogy accession register only mentions a skin for this specimen, raising the possibility that the skull (which confirmed the identity of the specimen beyond doubt to Thomas) had been retained on loan by Thomas at South Kensington, perhaps to be described one day, and was only reunited with the skin on its permanent arrival to the BMNH in 1939. Importantly, the Tunney *Zaglossus* specimen bears a third tag, added to the right hind leg either at the Tring Museum or when the specimen arrived with the Rothschild Bequest (Figure 9). This tag disputes the Tunney association of the specimen, noting, “other label apparently does not belong to this specimen” on one side, and “*Zaglossus bruijni goodfellowi* see Nov. Zoologicae vol 20, 1913” on the other side. The author of this label has been identified as Fred Young, who was a taxidermist at the Tring Zoological Museum, by Effie Ward, Tring librarian. The 1913 paper mentioned on this tag (Rothschild 1913) discusses *Zaglossus* specimens at BMNH and Tring that Rothschild was aware of in 1913, and lists them by taxon, age, sex, and preparation. The Tunney *Zaglossus* specimen, being an immature male skin (and, possibly, extracted skull) is not clearly associable with any specimens identified in this paper. The paper in question provides a taxonomic key for *Zaglossus* identification, and we take the tag’s reference to this *Novitates Zoologicae* paper to refer to this key. In effect, our understanding is that the person who wrote the message on this third tag resorted to using Rothschild’s key, and discovered that the Tunney specimen keys out to Rothschild’s concept of “*Zaglossus bruijnii goodfellowi”*, a taxon then considered endemic to the island of Salawati (= Salwaty, a continental island off the coast of western New Guinea)in Rothschild’s taxonomic scheme. We suggest that, on account of this specimen’s keyed identification, the writer of this last skin tag seems to have discounted the possibility that Tunney’s original tag details could be correct, and that this was done without any critical examination of the background and data associated with this specimen or consideration of Thomas’ extraction and examination of the skull and forelimb.

From the beginning of our investigations regarding this specimen, we have of course considered whether its original Tunney field tags truly belong to it, or whether they might have been transferred to it by mistake, as the latest tag associated with the specimen implies. However, several lines of evidence point to the fact that Tunney’s tags were always associated with an echidna, and that this tag was not likely to have been transferred by mistake from a *Tachyglossus* specimen to a *Zaglossus* specimen.

In addition to Tunney’s original tag, two sources—correspondence between Perth and London/Tring, and several parasitological publications—establish that Tunney’s specimen (his field number 347) was definitely an echidna, such that we are certain that its original tags were not transferred by mistake from a specimen of some other kind of animal. The specimen was mentioned in the original export paperwork, and discussed in parasitological literature, as *Tachyglossus aculeatus* (originally as *Echidna aculeata*), and its tag data, including the difficulty of sexing and the style of measurement, suggest an echidna. Thus the only conceivable mix-up could involve a *Tachyglossus* specimen collected by Tunney, with tags that became disassociated from the original specimen, and later erroneously attached to a specimen of *Zaglossus bruijnii* that came from New Guinea. However, we believe that Tunney’s original tags from Mt. Anderson are authentically associated with this *Zaglossus* specimen for several reasons. First, the nature and timing of any putative specimen switch is difficult to understand. Tunney collected only a few *Tachyglossus* during his expeditions in northern Australia, and these seem to be accounted for in the WAM and BMNH collections, and we note with interest that these tags were written somewhat differently. For example, on the tag of the only Tunney-collected *Tachyglossus* at BMNH, Tunney provided the name of the species as “Echidna” (left blank on the *Zaglossus* tag), and stated its abundance as “numerous” (“Rare” in the case of the *Zaglossus* specimen). Second, such a switch would have to have taken place after the echidna specimen arrived at Tring (in 1903-1904), not earlier in Perth, because no *Zaglossus* specimen was available in Perth—Tunney never collected in New Guinea, and the WAM has apparently never had a modern *Zaglossus* specimen in their mammal collection (as judged by details from the WAM accession register). But any switch must have already happened by the time that Thomas first inspected the Tunney specimens sent to Tring, as it seems clear that Thomas accepted that Tunney’s specimen number 347 was a *Zaglossus* collected in the Kimberley region once he was able to make confirming examinations of its skull and forelimb. Thomas had already published one report on Tunney’s 1903-1904 shipment to Tring by 1904 (Thomas 1904a), indicating that any switch that was unbeknownst to Thomas must have occurred at the very point of arrival at Tring. This is not impossible, but it is very difficult to imagine, especially in light of Rothschild’s clearly very active interest in all incoming echidnas. The Tunney skin came with two original tags, a small tag with field number wired to the leg, and a heavier card tag tied to a leg with strong twine. Both tags would need to have been removed from a *Tachyglossus* specimen, and reattached by mistake to one of Rothschild’s few and precious *Zaglossus* skins at Tring, which is unlikely. A decade later, there were still only 13 *Zaglossus* specimens identified in the Tring collection, and it is clear from Rothschild’s publications that these were highly valued and carefully curated by Rothschild (Rothschild 1913). In summary, it is highly implausible to envision a switch-up in Perth or in Tring that could explain how tags from an Australian *Tachyglossus* specimen would have become mistakenly associated with a specimen of *Zaglossus* that originated from New Guinea.

Another important consideration is the size of the animal measured by Tunney. Tunney’s tag gives the specimen’s total length measurement as 21 inches (= 533 mm), and this value matches very well the size of the study skin to which it is currently attached, as measured with a flexible measuring tape. This body size measurement is consistent with either a subadult *Zaglossus* (i.e., like the specimen to which it is attached) or an unusually large adult *Tachyglossus*. Total length measurements of 539-1000 mm have been reported for adult *Zaglossus bruijnii* (Allen 1912, Rothschild 1913). Extremely large *Tachyglossus* specimens only rarely reach the lower limit of this size range. Typical lengths for adult *Tachyglossus* are in the range of 300-450 mm (Wood Jones 1923, Menkhorst and Knight 2001, Augee 2005). An unusually large and aged male *Tachyglossus aculeatus* (USNM 283961) from Groote Eylandt, Northern Territory, measuring 555 mm in total length (original field measurements), approximates a maximum body size for the genus. This specimen is by far the largest *Tachyglossus* in the USNM collection (of about 40 specimens), and is among the very largest and most robust of *Tachyglossus* specimens in world museums by skin and skull size (K. Helgen and G. Perri, in litt, 2012). In summary, only the largest *Tachyglossus* outliers on record could match the size of the echidna reported on Tunney’s tag, Tunney’s measurements are consistent with the dimensions of a *Zaglossus* nearing cranial maturity, and Tunney’s measurements are a match for the actual dimensions of the specimen to which his tags are attached.

### The specimen’s locality

The tag locality provided by Tunney for the *Zaglossus* specimen is “Mt. Anderson, W Kimberley.” First named in 1879 by Alexander Forrest in his “North-West Expedition” from DeGrey to Darwin (Forrest 1880), Mount Anderson is situated near the Grant Ranges, along the Fitzroy River about 90 km southeast of Derby at approximately 18^o^02'S, 123^o^56'E (Storr 1966). In early October, Tunney sailed from Port Hedland to Derby, where he arrived about October 11, and he collected at and in the vicinity of Mount Anderson from the end of October to late November (Storr 1966), during which time (20 November 1901) the echidna was collected.

Inland areas of the West Kimberley were settled by white Australians for sheep and cattle pastoralism in the aftermath of Forrest’s surveys (since 1881) but the region has historically been very sparsely inhabited by both European and Aboriginal communities (Bolton 1952, Speck 1964) and remains so today. Tunney’s visit to the area took place in the early decades of the region’s utilization for livestock. It was the first reconnaissance for mammals in this region, and the immediate area remains zoologically little known today. On its tag, Tunney characterized the habitat of the *Zaglossus* specimen as “conglomerate hills”, suggesting that the animal was found in a rocky area, where echidnas would surely make their burrows. The only other mammals obtained by Tunney at Mount Anderson were specimens of the large macropodids *Megalibgwilia antilopinus* and *Megalibgwilia robustus* (specimens at BMNH). Birds collected by Tunney at Mount Anderson included the Varied Lorikeet (*Psitteuteles versicolor*), Red-backed Fairywren (*Malurus melanocephalus cruentatus*), and Brown Goshawk (*Accipiter fasciatus cruentus*) (specimens in the AMNH ornithological collections).

The distribution of *Zaglossus* in New Guinea is today centered on montane tropical rainforests (but open areas of subalpine grassland are also prime habitat, and some areas of lowland forest and limestone country are also utilized). It might thus be expected that the last areas of survival for *Zaglossus* populations in the Kimberley would be in the region’s many tiny and scattered evergreen rainforest fragments, which are largely distributed to the north of the Fitzroy River (McKenzie et al. 1991). However, the Mount Anderson area is reasonably well watered (Registrar et al. 1902), and its inaccessibility, sparse human population, and the availability of rugged, steep, rocky areas may explain its importance in hosting a late-surviving remnant population of *Zaglossus* in Australia. Six decades after Tunney’s visit, in their review of “Lands of the West Kimberley Area”, Speck et al. (1964: 191) discussed the Grant Ranges-Mt. Anderson area under their classification of “inaccessible country” and “inaccessible pasture land”, describing it thus:

"*Environment. The rugged mountain ranges, elevated plateaux, steep hills, and associated valleys have a complex geological pattern with quartzites, sandstones, shales, slates, schists, basalt, dolerite, and limestone. It is mostly rough, inaccessible, unproductive, and undeveloped. Soils are varied but characteristically skeletal with extensive outcrop*.

*Composition. Most of the lands are within the higher-rainfall area and the vegetation of these parts is an open woodland with moderate shrub layer and grassy ground storey of curly spinifex pasture type…. In the lower-rainfall parts the vegetation is more stunted and open and the grass layer is hard spinifex… Grasses other than spinifex are poorly represented. Edible top feed is also scanty*.

*Pastoral Value. Only where these lands are adjacent to better country is utilization possible. They are more likely to have a nuisance value. They are generally well watered and therefore provide a hideout for scrub bulls, increasing the difficulty of herd management and mustering. At best it will remain extremely poor pastoral country*.

*Reaction to Grazing, and Management. Much of the area is unstocked and there is little or no evidence of pasture degradation or denudation except in isolated, restricted areas adjacent to watering points*."

A visual representation of the vegetation currently present around Mount Anderson today can be seen with mapping resources available in the online resource *Atlas of Living Australia* (http://spatial.ala.org.au/), which indicates that present vegetation is dominated by “Acacia open woodlands” but also includes some small areas of “Rainforest and vine thickets.” We suggest that these latter habitats (rainforest, vine thickets) would be relevant remnant habitat for *Zaglossus*, and that these habitats were likely more expansive at the time of Tunney’s visit to the region well over a century ago in 1901.

Relatively inaccessible and sparsely inhabited rocky areas provide some of the most important remaining areas of occurrence for *Zaglossus* in New Guinea, on the southern and northern slopes of the Central Cordillera, and in limestone country throughout the “Bird’s Neck” region in the west of the island. That similarly remote and sparsely inhabited areas of northern Australia apparently sheltered at least one remnant population of *Zaglossus* into the twentieth century is an astonishing realization, and serves as strong encouragement for wildlife researchers to undertake surveys of remote candidate areas of northern Australia with the goal of establishing whether *Zaglossus* may still exist in any rainforest fragments or rugged gorges across the Kimberley.

### Taxonomy, biogeography, and the Kimberley *Zaglossus*

We confidently identify the Kimberley specimen of *Zaglossus* as the Western Long-Beaked Echidna, *Zaglossus bruijnii*, otherwise known only from the western portion of the island of New Guinea, which it matches in size, cranial features, claw number, and pelage features. *Zaglossus bruijnii* is the only echidna taxon that typically lacks claws on the first and fifth digits of all feet (the claw conformation seen in the Kimberleyspecimen), and always lacks a claw on the first digit of the hindfeet (a claw is always present on the first digit of the hindfoot in *Zaglossus bartoni*) (Flannery and Groves 1998). As Oldfield Thomas noted, the subadult skull of the Kimberley specimen is a good match in overall size and shape for specimens of *Zaglossus bruijnii* that are of equivalent age (as judged by cranial development, in terms of robustness and sutural ossification). The relatively short and modest (rather than long and heavy) growth of fur in between the spines on the dorsum, relatively pale brown (rather than blackish brown) pelage, presence of some black-tipped spines on the dorsum, and presence of visible spines on the underside, are external features that in combination are typical only of lowland populations of *Zaglossus bruijnii*, such as those recorded from the land-bridge island of Salawati, the adjacent Vogelkop coast at Sorong, and the Fakfak and Charles Louis Ranges in the Bird’s Neck region (skins especially at the BMNH, MCZ, and MZB).

The Western Long-beaked echidna, *Zaglossus bruijnii*, occurs in western New Guinea in habitats from as low as sea level up to the top of the highest peaks in the Vogelkop Peninsula—in the Tamrau and Arfak Ranges (to 2900 m)—and from the land-bridge island of Salawati in the west, to the “Bird’s Neck region” of New Guinea in the east, extending as far east as the Fakfak Range and possibly the Charles Louis Ranges on the western edge of the Central Cordillera (in the south) and possibly to the eastern shores of Geelvink (= Cenderawasih) Bay (in the north) (Rothschild in Thomas and Rothschild 1922, Van Deusen and George 1969, Flannery 1995a, 1995b, Flannery and Groves 1998, Aplin et al. 1999, Helgen 2007). The Eastern long-beaked echidna, *Zaglossus bartoni*, does not usually occur in habitats below about 1000 m, with low-elevation records known only in far south-eastern New Guinea at Collingwood Bay (down to 0–200 m) and in the vicinity of Haia on the south side of the Central Cordillera in east-central New Guinea (down to about 500–600 m).

Though previously recorded only from western New Guinea, *Zaglossus bruijnii* is the *Zaglossus* taxon occurring in closest geographical proximity to the Kimberley region, and is the only *Zaglossus* regularly documented in lowland contexts. Given that similar relevant habitats, including sparsely inhabitated limestone country and remnant rainforests, are to be found across the Kimberley region, it does not surprise us that the modern Kimberley representative of the genusshould be *Zaglossus bruijnii*. We envision a late Pleistocene distribution of *Zaglossus bruijnii* that extended across rugged, rocky country and rainforests along the western parts of the Sahul Shelf, comprising much of the area between Australia and New Guinea that has been inundated by the Arafura Sea since the terminal Pleistocene, thus connecting the restricted Recent range from the Vogelkop Peninsula in the north to the Kimberley region and Arnhem Land in the south.

### Ectoparasites from the Kimberley *Zaglossus*

The Kimberley *Zaglossus* specimen, while overlooked in mammalogical literature, has been referenced with surprising regularity in parasitological papers. It is the “symbiotype” (i.e., host to the type series; Frey et al. [1992]) for two ectoparasite taxa, the flea *Echidnophaga liopus* Jordan and Rothschild, 1906; and the ixodid tick *Amblyomma australiense* Neumann, 1905. In all of the parasitological literature in which Tunney’s specimen is referenced, the specimen is mistakenly referred to as a *Tachyglossus* (e.g., Neumann 1905, Jordan and Rothschild 1906, Robinson 1926, Roberts 1953, Dunnet and Mardon 1974, Keirans 1982), obscuring until now the correct host association of these parasites.

The flea *Echidnophaga liopus* is so far documented firmly only from Tunney’s *Zaglossus* specimen and is unknown to date in *Tachyglossus*. Other *Echidnophaga* specimens attributed to *Echidnophaga liopus* in literature, which come from Indian rodents (Rothschild and Jordan 1906), seem more likely to represent a distinct Asian species (Dunnet and Mardon 1974). Out of interest, we note that two of the most commonly recorded and host-specific fleas of *Tachyglossus aculeatus*—*Echidnophaga ambulans* and *Bradiopsyllae echidnae* (Dunnet and Mardon 1974)—have not been recovered from Tunney’s specimen.

Neumann (1905) originally described the Australian tick *Amblyomma australiense* based on examplars in the N.C. Rothschild collection taken from Tunney’s *Zaglossus* specimen (Robinson 1926, Roberts 1964, Keirans 1982). Apart from this original record from Tunney’s *Zaglossus*, a few other records of occurrence are available for this apparently northern Australian tick; Taylor (1913) and Roberts (1964) recorded it from *Tachyglossus* at Townsville in Queensland, Robinson (1926) recorded it from a unidentified large lizard at Darwin in Northern Territory, and Roberts (1964) recorded it from an “unspecified snake”, also at Darwin. The closely related tick *Amblyomma echidnae* Roberts, 1953, considered by some to be conspecific with *Amblyomma australiense* (but held as distinct, pending critical study, by Guglielmone et al. [2009]) is apparently known to date only from Australian *Tachyglossus* (Roberts 1953, 1964, 1970).

So far, none of the ectoparasites recorded from Tunney’s *Zaglossus* have been reported from New Guinea *Zaglossus*, but very little is known about the parasites of Long-beaked echidnas. We are aware of only two ectoparasites definitively recorded from New Guinea *Zaglossus*. The tick *Bothriocroton oudemansi* (Neumann, 1910) has been reported from *Zaglossus bruijnii* at Fakfak, and from *Zaglossus bartoni* in the Central Cordillera (Beati et al. 2008). The tick *Ixodes zaglossi* Kohls, 1960, was described from a specimen of *Zaglossus bartoni* from the Wissel Lakes at the western end of New Guinea’s Central Cordillera. Whether either of these ticks is the same taxon as *Ixodes acanthoglossi* Lucas, 1878 (regarded as an indeterminable *nomen dubium* by Beati et al. [2008]), originally described as a parasite of *Zaglossus bruijnii* in the Arfak Mountains, has not been determined.

### Possible Aboriginal familiarity with *Zaglossus* in the Kimberley

The Late Quaternary occurrence of Long-beaked echidnasin northern Australia is widely accepted on the basis of a compelling Aboriginal rock art illustration (Figure 2), from an undisclosed Arnhem Land locality, that accurately depicts *Zaglossus* (Murray and Chaloupka 1984). This illustration, usually considered Late Pleistocene in age, has been often reproduced in reference books on Australian mammals (e.g. Johnson 2006, Tyndale-Biscoe 2005), and demonstrates Australian Aboriginal familiarity and interaction with Long-beaked echidnas.

It is possible that Aboriginal Australians also interacted with *Zaglossus* much more recently. In 2001, years before we became aware of the Australian provenance of the *Zaglossus* specimen reported here, one of us (Kohen) recorded a potential example of living memory of *Zaglossus* while engaged in field work in the East Kimberley. His account of the experience is as follows:

*While conducting faunalsurveys at Faraway Bay, I was accompanied by an Aboriginal woman in her fifties who belonged to the Miriwoong Gadjerong tribe. Their territory extends from the coast inland in the region close to the Western Australia-Northern Territory border. In this part of Australia, tribal affiliation is passed down through the female line. However, Faraway Bay is on her father’s country, and he belonged to the Kwini tribe*.

*While walking close to the coast, we found a scat. On asking my informant what she thought it was, she correctly identified it as an echidna scat, which she referred to as “porcupine”. As only one echidna is traditionally known from Australia, I assumed that it belonged to* Tachyglossus.* A few hours later we had returned to the camp and were sharing tea when she commented about the echidna scat we had found. She said that her grandmothers “used to hunt the other one”. I asked her what other one, and she said that she meant a much larger echidna. She indicated its height which I estimated to be around 40 cm*.

*I was intrigued, as both of her grandmothers were still alive and in their nineties. However, one had recently suffered a stroke and the other lived some distance away. When we returned to Kununurra, I had an opportunity to speak to my informant’s mother. As it happened, I had a copy of Tim Flannery’s 1990 paper* [Flannery 1990] *on the extinct megafauna of Australia, which included a series of shadow illustrations of large Australian mammals. When I showed this to her, and asked her if she knew any of these animals, she identified the* Zaglossus.* My impression was that the animal had not been seen for a long time*.

We readily acknowledge that these kinds of informant accounts are fraught with difficulty of interpretation. However, we mention these interactions, because, like the Tunney specimen, this information from Kohen’s informants could be relevant to the survival of *Zaglossus* in the Kimberley region into the twentieth century. We suggest that future efforts to investigate the recent survival of *Zaglossus* in remote northern Australia take into account evidence that may be derived from cultural sources such as rock art, living memories from Aboriginal cultures, and examination of vocabularies relevant to animal names in Aboriginal languages.

## Discussion

### Specimen-based evidence of recent survival of *Zaglossus* in Australia

We are sufficiently convinced by the tags and information associated with the Tunney *Zaglossus* specimen to regard it as evidence for the survival of the long-beaked echidna in the Kimberley region into the early twentieth century. We accordingly recommend that the Western Long-beaked echidna, *Zaglossus bruijnii*, be included in future faunal compilations of the modern mammal fauna of Australia (e.g., Walton 1988, Walton and Richardson 1989, Menkhorst and Knight 2001, Van Dyck and Strahan 2005), and on the long list of mammal species that have declined to extirpation, extinction, or near extinction in Australia over the past two centuries (Johnson 2006).

We realize that, despite our conclusions, summarized here, others may remain skeptical of this *Zaglossus* specimen’s association with Tunney’s tags. Additional studies of this remarkable specimen might include analyses of ancient DNA, stable isotopes, and trace elements to test its origins and the context of its collection. Further targeted studies of relevant Kimberley Pleistocene and Holocene subfossil assemblages (e.g. O’Connor et al. [2008], Start et al. [2012]) may also shed useful light on the late survival of *Zaglossus* in northern Australia.

### Quaternary and recent extinctions

Most of Australia’s remarkable Pleistocene megafauna (gigantic marsupials, reptiles, and birds) became extinct after about 50,000 years before the present (Flannery 1994, Roberts et al. 2001, Miller et al. 2005, Rule et al. 2012) following the arrival of humans to the continent. Many other mammal species declined broadly, many to the point of extinction, across their mainland Australian distributions during the Holocene (e.g., thylacine, *Thylacinus*; Tasmanian devil, *Sarcophilus*) or since the onset of European impacts in the mid-1800s (Flannery 1994, Johnson 2006). The Tunney specimen adds *Zaglossus bruijnii* to the list of mammal species that underwent dramatic declines in Australia during recent times.

Anotherrather unexpected recent addition to the list of Quaternary extinctions in the Kimberley region is a fruit-bat of the genus *Styloctenium*, identified by Pettigrew et al. (2008) from definitive illustrations in (Late Pleistocene?) rock art from near Kalumburu. Species of *Styloctenium* are today known only from the large Indonesian island of Sulawesi (and some of its satellites) and the Philippine island of Mindoro (Bergmans and Rozendaal 1988, Esselstyn 2007). We also note with interest that compelling rock art images of the large, extinct carnivorous marsupial *Thylacaleo* have also recently come to light in the Kimberley (Akerman 2009, Akerman and Willing 2009). These discoveries point to the importance of rock art as a source of information about the past distributions in time and space of animals in northern Australian contexts.

Both *Styloctenium* and *Zaglossus* are largely rainforest-associated lineages that today are known only from tropical islands north of Australia. Their presence in the late Quaternary fauna of the Kimberley region doubtless reflects the former presence of extensive mesic forested habitats across much of northern Australia, with fragmentation and extinction of forest-reliant species driven by a combination of climate-change and prehistoric human impacts in recent millennia (McKenzie et al. 1991)—a topic that so far has received much greater attention in the Wet Tropics of Queensland than in the tropical rainforests of the Kimberley region (Joseph et al. 1995, Williams 1997, Schneider and Moritz 1999, Bowman et al. 2010). Today, none of the mammal species known to occur in Kimberley rainforests are entirely dependent on rainforest habitats (Friend et al. 1991). However, we suggest that further critical examination of rock art depictions, and of fossil and subfossil assemblages, will likely illuminate the former presence in the Kimberley rainforests of additional taxa typical of modern Indo-Malayan or Australo-Papuan rainforest habitats.

We hold out a small optimism that Long-beaked echidnasmight yet dig burrows and hunt invertebrates in at least one hidden corner of Australia’s north-west. Such hopes are founded on the remoteness of this little-studied expanse of the Australian continent, and on the relatively late discovery of other medium-sized Kimberley mammals including the Monjon, *Petrogale burbidgei* (Kitchener and Sanson 1978), a small rock wallaby endemic to the north-west Kimberley, and the recent rediscovery of the Scaly-tailed Possum (*Wyulda squamicaudata*) in the eastern Kimberley (Doody et al. 2012), where it had not been recorded since 1917.

All living *Zaglossus* taxa in New Guinea are considered to be critically endangered (Isaac et al. 2007, Leary et al. 2008a, 2008b, 2008c), with primary threats being subsistence hunting and habitat loss. While it is vitally and urgently important to explore whether *Zaglossus* still survives in remote areas of the Kimberley, it is also important to learn more about its recent distribution and history of decline. Such studies may identify hitherto unsuspected dimensions to the adaptive breadth of *Zaglossus* species, reveal the existing or former presence of populations with genetic diversity unrepresented in New Guinea, and possibly assist with long term conservation of *Zaglossus* in New Guinea by illuminating important patterns of habitat occurrence and historical decline in Australia. If *Zaglossus bruijnii* is extinct in the Kimberley region, it might also warrant consideration as a candidate for reintroduction, once more is learned of its former distribution and ecological role.

### Ecology of *Zaglossus bruijnii* in western New Guinea and Australia

Little is definitively recorded about diet in Long-beaked echidnas. Most information is based on anecdotes or extremely limited studies of *Zaglossus bartoni*, which is thought to be a specialist earthworm feeder that also feeds on subterranean arthropods including centipedes and large insect larvae (Griffiths 1978, Griffiths et al. 1991, Flannery 1995a, Opiang 2009), with no evidence for feeding on social insects such as ants and termites, the principal diet of *Tachyglossus* (e.g. Abensperg-Traun and De Boer 1992). Because of the intimate cranial resemblance between *Zaglossus bartoni* and other *Zaglossus* taxa, it can be expected that the diets of these congeners are similar. However, it is possible that *Zaglossus bruijnii* eats more social insects than *Zaglossus bartoni*, perhaps especially in lowland environments. One of only two comments about diet in an individual of *Zaglossus bruijnii* is the firsthand account of naturalist Thomas Barbour, recounted by Allen (1912:302):

*This specimen was kept alive for about a month and a few observations on its habits were made. It was absolutely nocturnal and spent the day partially buried in the deep layer of sand which was kept in its cage… At night it moved about sluggishly, often digging with motions that strongly recalled those of a turtle. It fed on ants only, which were procured by placing in a dish a considerable amount of shredded cocoanut. The ants soon swarmed in this and the whole was then placed in the Proechidna’s cage. It ate the insects by thrusting its long tongue down into the cocoanut. It took a little water or water with condensed milk, but seemed to drink very little*.

It may of course be the case that this animal only ate ants because other, more favored foods were not offered to it. Ripley (1942: 256-259), provided the only other (conflicting) account that references the diet of *Zaglossus bruijnii*, based on a captive animalat Sansapor (a settlement adjacent to the Tamrau Range), noting “The echidna is supposed to live on ants, although this one would never touch them, much preferring papaya and raw eggs.” Studies of the anatomy of the salivary glands of *Zaglossus bruijnii* (not available for *Zaglossus bartoni*) show these glands to be greatly developed, as in various ant-eating animals, and very similar to those of *Tachyglossus* (Viallanes 1880, Allen 1912). Ants and termites would presumably constitute a reliable source of food for a large echidna in northern Australian contexts (Andersen and Majer 1991, Milewski et al. 1994, Barrow et al. 2006, 2007), though earthworms might remain key resources especially in remnant rainforest areas (McKenzie and Dyne 1991). If *Zaglossus bruijnii* is more dependent on ants in lowland New Guinea (and Australian) habitats than other *Zaglossus* populations arein montane New Guinea habitats, its presence across northern Australia until recent times may shed new light on the riddle, posed by Milewski et al. (1994), of why, unlike other southern continents, Australia seems to have had no species larger than *Tachyglossus* exploiting social insects as a major food resource. Griffiths et al. (1991) similarly suggested that *Megalibgwilia*, the other (and more southerly distributed) large echidna genus present in the Australian Quaternary, may also have been an insect-eater rather than an earthworm-eating specialist.

## References

[B1] Abensperg-Traun M De Boer ES (1992) The foraging ecology of a termite-and ant-eating specialist, the echidna *Tachyglossus aculeatus* (Monotremata: Tachyglossidae). Journal of Zoology (London) 226: 243-257. doi: 10.1111/j.1469-7998.1992.tb03837.x

[B2] Akerman K (2009) Interaction between humans and megafauna depicted in Australian rock art? Antiquity 83(322). http://www.antiquity.ac.uk/projgall/akerman322/

[B3] Akerman K Willing T (2009) An ancient rock painting of a marsupial lion, *Thylacaleo carnifex*, from the Kimberley, Western Australia. Antiquity 83(319). http://www.antiquity.ac.uk/projgall/akerman319/

[B4] Allen GM (1912) *Zaglossus*. Memoirs of the Museum of Comparative Zoology 40: 253-307.

[B5] Andersen AN Majer JD (1991) The structure and biogeography of rainforest ant communities in the Kimberley region of northwestern Australia. In: McKenzie NL Johnston RB Kendrick PG (Eds). Kimberley rainforests of Australia. Surrey Beatty and Sons, Chipping Norton, New South Wales: 333-346.

[B6] Aplin KP Pasveer JM Boles WE (1999) Late Quaternary vertebrates from the Bird’s Head Peninsula, Irian Jaya, Indonesia, including descriptions of two previously unknown marsupial species. Records of the Western Australian Museum Supplement 57: 351-387.

[B7] Augee ML (2005) Short-beaked echidna, *Tachyglossus aculeatus* (Shaw, 1792). In: Van Dyck S Strahan R (Eds). The mammals of Australia. Third edition. Reed New Holland, Sydney: 37-39.

[B8] Baillie JEM Turvey ST Waterman C (2009) Survival of Attenborough’s long-beaked echidna *Zaglossus attenboroughi* in New Guinea. Oryx 43: 146-148. doi: 10.1017/S0030605309002269

[B9] Barrow L Parr CL Kohen JL (2006) Biogeography and diversity of ants in Purnululu (Bungle Bungle) National Park and Conservation Reserve, Western Australia. Australian Journal of Zoology 54: 123-136. doi: 10.1071/ZO06009

[B10] Barrow L Parr CL Kohen JL (2007) Habitat type influences fire resilience of ant assemblages in the semi-arid tropics of northern Australia. Journal of Arid Environments 6: 80-95. doi: 10.1016/j.jaridenv.2006.08.005

[B11] Beati L Kerians JE Durden LA Opiang MD (2008) *Bothriocroton oudemansi* (Neumann, 1910) n. comb. (Acari: Ixodida: Ixodidae), an ectoparasite of the western long-beaked echidna in Papua New Guinea: redescription of the male and first description of the female and nymph. Systematic Parasitology 69: 185-200. doi: 10.1007/s11230-007-9115-5 18210218

[B12] Bergmans W Rozendaal RG (1988) Notes on collections of fruit bats from Sulawesi and some off-lying islands (Mammalia: Megachiroptera). Zoologische Verhandelingen 248: 1-74.

[B13] Bolton GC (1952) A survey of the Kimberley pastoral industry from 1885 to present. MSc Thesis, University of Western Australia, Perth, Australia.

[B14] Bowman DMJS Brown GK Braby MF Brown JR Cook LG Crisp MD Ford F Haberle S Hughes J Isagi, Y Joseph L McBride J Nelson G Ladiges PY (2010) Biogeography of the Australian monsoon tropics. Journal of Biogeography 37: 201-216. doi: 10.1111/j.1365-2699.2009.02210.x

[B15] Chadwick R (2008) ‘Your obedient servant’: the John Tunney collection at the Western Australian Museum. In: Peterson N Allen L Hamby L (Eds). The makers and making of indigenous Australian museum collections. Melbourne University Press, Victoria: 255-280.

[B16] Chaloupka G Murray P (1986) Dreamtime or reality? Reply to Lewis. Archaeology in Oceania 21: 145-147.

[B17] Coues E (1895) The genus *Zaglossus*. Science (n.s.) 1: 610. 10.1126/science.1.22.610 17806827

[B18] Doody JS Rhind D Castellano CM Bass M (2012) Rediscovery of the scaly-tailed possum (*Wyulda squamicaudata*) in the eastern Kimberley. Australian Mammalogy 34: 260-262. doi: 10.1071/AM11039

[B19] Dunnet GM Mardon DK (1974) A monograph of Australian fleas (Siphonaptera). Australian Journal of Zoology Supplementary Series 30: 1-273.

[B20] Esselstyn JA (2007) A new species of stripe-faced fruit bat (Chiroptera: Pteropodidae: *Styloctenium*) from the Philippines. Journal of Mammalogy 88: 951-958. doi: 10.1644/06-MAMM-A-294R.1

[B21] Flannery T (1990) Pleistocene faunal loss: implications of the aftershock for Australia’s past and future. Archaeology in Oceania 25: 45-67.

[B22] Flannery TF (1994) The Future Eaters: an ecological history of the Australasian lands and people. Reed Books, Sydney.

[B23] Flannery TF (1995a) Mammals of New Guinea. Revised edition. Reed Books, Chatswood, New South Wales.

[B24] Flannery TF (1995b) Mammals of the South-west Pacific and Moluccan Islands. Reed Books, Chatswood, New South Wales.

[B25] Flannery TF Groves CP (1998) A revision of the genus *Zaglossus* (Monotremata, Tachyglossidae), with description of new species and subspecies. Mammalia 62: 367-396. doi: 10.1515/mamm.1998.62.3.367

[B26] Forrest A (1880) North-West exploration: journal of expedition from DeGrey to Port Darwin. Richard Pether, Government Printer, Perth.

[B27] Frey JK Yates TL Duszynski D Gannon WL Gardner SL (1992) Designation and curatorial management of type host specimens (symbiotypes) for new parasite species. Journal of Parasitology 78: 930-932. doi: 10.2307/3283335

[B28] Friend GR Morris KD McKenzie NL (1991) The mammal fauna of Kimberley rainforests. In: McKenzie NL Johnston RB Kendrick PG (Eds). Kimberley rainforests of Australia. Surrey Beatty and Sons, Chipping Norton, New South Wales: 393-412.

[B29] Gervais P (1877a) L’échidné de la Nouvelle-Guineé. Comptes Rendus des Séances de L’Académie des Sciences 85: 837-838.

[B30] Gervais P (1877b) L’échidné de la Nouvelle-Guineé. Deuxième Note. Comptes Rendus des Séances de L’Académie des Sciences 85: 990-991.

[B31] Gill T (1877) Vertebrate zoology. In: Baird SF (Ed.). Annual record of science and industry for 1876. Harper and Brothers, New York: 171-172.

[B32] Gill T (1885) The species of tachyglossids. Annual Report of the Board of Regents of the Smithsonian Institution 1884: 642-643.

[B33] Glauert L (1914) Taxonomy and detailed description of *Zaglossus hacketti*. Records of the Western Australian Museum 1: 244-248.

[B34] Gray V (2003) A pair of every species. In: Rothschild E (Chair) The Rothschild Archive Trust. Rothschild Archive Trust, London, 46–50.

[B35] Griffiths M (1978) The biology of the monotremes. Academic Press, New York.

[B36] Griffiths M Wells RT Barrie DJ (1991) Observations on the skulls of fossil and extant echidnas (Monotremata: Tachyglossidae). Australian Mammalogy 14: 87-101.

[B37] Groves CP (2005) Order Monotremata. In: Wilson DE Reeder DR (Eds). Mammal species of the world: a taxonomic and geographic reference, Third edition. Johns Hopkins University Press, Baltimore, Maryland: 1-2.

[B38] Guglielmone AA Robbins RG Apanaskevich DA Petney TN Estrada-Pefia A Horak IG (2009) Comments on controversial tick (Acari: Ixodida) species names and species described or resurrected from 2003 to 2008. Experimental and Applied Acarology 48: 311-327. doi: 10.1007/s10493-009-9246-2 19169832

[B39] Hartert E (1905) List of birds collected in north-western Australia and Arnhem-Land by Mr. J.T. Tunney. Novitates Zoologicae 12: 194-242.

[B40] Helgen KM (2007) A taxonomic and geographic overview of the mammals of Papua. In: Marshall AJ Beehler B (Eds). The ecology of Papua (Ecology of Indonesia series Volume VI). Periplus Editions, Singapore: 689-749.

[B41] Hill JE (1990) A memoir and bibliography of Michael Roger Oldfield Thomas, F.R.S. Bulletin of the British Museum, Natural History (History Series) 18: 25-113.

[B42] Isaac NJB Turvey ST Collen B Waterman C Baillie JEM (2007) Mammals on the EDGE: conservation priorities based on threat and phylogeny. PLoS ONE 2(3): e296. doi: 10.1371/journal.pone.0000296. PMC1808424 17375184

[B43] Johnson C (2006) Australia’s mammal extinctions: a 50,000 year history. Cambridge University Press, Cambridge.

[B44] Jordan K Rothschild NC (1906) A revision of the Sarcopsyllidae, a family of Siphonaptera. Thompson Yates and Johnstone Laboratories Reports 7: 15-72.

[B45] Joseph L Moritz C Hugall A (1995) Molecular support for vicariance as a source of diversity in rainforest. Proceedings of the Royal Society Series B Biological Sciences 260: 177-182. doi: 10.1098/rspb.1995.0077 7784437

[B46] Keirans JE (1982) The tick collection (Acarina: Ixodoidea) of the Hon. Nathaniel Charles Rothschild deposited in the Nuttall and general collections of the British Museum (Natural History). Bulletin of the British Museum of Natural History (Zoology) 42 (1): 1-36.

[B47] Kitchener DJ Sanson G (1978) *Petrogale burbidgei* (Marsupialia, Macropodidae), a new rock wallaby from Kimberley, Western Australia. Records of the Western Australian Museum 6: 269-285.

[B48] Kohls GM (1960) *Ixodes (Endopalpiger) zaglossi* n. sp. from the long-beaked echidna of New Guinea (Acarina, Ixodidae). Acarologia 2: 447-452.

[B49] Krefft G (1868) On the discovery of a new and gigantic fossil species of *Echidna* in Australia. Annals and Magazine of Natural History (series 4) 1: 113–114.

[B50] Leary T Seri L Flannery T Wright D Hamilton S Helgen K Singadan R Menzies J Allison A James R Aplin K Salas L Dickman C (2008a)*Zaglossus attenboroughi*. IUCN Red List of Threatened Species. www.iucnredlist.org [Version 2012.2]

[B51] Leary T Seri L Flannery T Wright D Hamilton S Helgen K Singadan R Menzies J Allison A James R Aplin K Salas L Dickman C (2008b)*Zaglossus bartoni*. IIUCN Red List of Threatened Species. www.iucnredlist.org [Version 2012.2]

[B52] Leary T Seri L Flannery T Wright D Hamilton S Helgen K Singadan R Menzies J Allison A James R Aplin K Salas L Dickman C (2008c)*Zaglossus bruijnii*. IUCN Red List of Threatened Species. www.iucnredlist.org [Version 2012.2]

[B53] Lewis D (1986) ‘The dreamtime animals’: a reply. ArchaeologyinOceania 21: 140-145.

[B54] Long J Archer M Flannery T Hand S (2002) Prehistoric mammals of Australia and New Guinea: 100 million years of evolution. University of New South Wales Press, Sydney.

[B55] Lucas H (1878) Note. Annales de la Société Entomologique de France 8: 35-36.

[B56] Mahoney JA Ride WDL (1975) Index to the genera and species of fossil Mammalia described from Australia and New Guinea between 1838 and 1969. Western Australian Museum Special Publication 6: 1-247.

[B57] McKenzie NL Belbin L Keigherry GJ Kenneally KF (1991) Kimberley rainforest communities: patterns of species composition and Holocene biogeography. In: McKenzie NL Johnston RB Kendrick PG (Eds). Kimberley rainforests of Australia. Surrey Beatty and Sons, Chipping Norton, New South Wales: 423-452.

[B58] McKenzie NL Dyne GR (1991) Earthworms of rainforest soils in the Kimberley, Western Australia. In: McKenzie NL Johnston RB Kendrick PG (Eds). Kimberley rainforests of Australia. Surrey Beatty and Sons, Chipping Norton, New South Wales: 133-144.

[B59] McKenzie NL Johnston RB Kendrick PG (1991) Kimberley rainforests of Australia. Surrey Beatty and Sons, Chipping Norton, New South Wales.

[B60] Menkhorst P Knight F (2001) A field guide to the mammals of Australia. Oxford University Press, South Melbourne.

[B61] Milewski AV Abensperg-Traun M Dickman CR (1994) Why are termite- and ant-eating mammals smaller in Australia than in southern Africa: history or ecology? Journal of Biogeography 21: 529–543. doi: 10.2307/2845656

[B62] Miller GH Fogel ML Magee JW Gagan MK Clarke SJ Johnson BJ (2005) Ecosystem collapse in Pleistocene Australia and a human role in megafaunal extinction. Science 309: 287–290. 10.1126/science.1111288 16002615

[B63] Murray P (1978a) Late Cenozoic monotreme anteaters. In: Augee ML (Ed). Monotreme biology. Royal Zoological Society of New South Wales, Taronga Zoo, Mosman, New South Wales: 29-55.

[B64] Murray P (1978b) A Pleistocene spiny anteater from Tasmania (Monotremata: Tachyglossidae, *Zaglossus*). Papers and Proceedings of the Royal Society of Tasmania 112: 39-68.

[B65] Murray P Chaloupka G (1984) The dreamtime animals: extinct megafauna in Arnhem Land rock art. Archaeology in Oceania 19: 105-116.

[B66] Neumann LG (1905) Notes sur les Ixodidés—III. Archives de Parasitologie 9: 225-241.

[B67] Neumann LG (1910) Description de deux nouvelles espèces d’Ixodinae. Tijdschrift voor Entomologie 53: 11-17.

[B68] O’Connor S Aplin K Collins S (2008) Results of a small salvage excavation in Windjana Gorge, Kimberley, Western Australia. Archaeology in Oceania 43: 75-88.

[B69] Opiang MD (2009) Home ranges, movement, and den use in long-beaked echidnas, *Zaglossus bartoni*, from Papua New Guinea. Journal of Mammalogy 90: 340-346. doi: 10.1644/08-MAMM-A-108.1

[B70] Owen R (1884) Evidence of a large extinct monotreme (*Echidna ramsayi* Owen) from the Wellington Breccia Cave New South Wales. Philosophical Transactions of the Royal Society of London 185: 273-275. doi: 10.1098/rstl.1884.0013

[B71] Palmer TS (1895) The generic names of the three-toed echidna. Science 1: 518. 10.1126/science.1.19.518 17833680

[B72] Peters W Doria G (1876) Descrizione di una nuova specie di *Tachyglossus* proveniente dalla Nuova Guinea settentrionale. Annali del Museo Civico Storia Naturale di Genova 9: 183–185.

[B73] Pettigrew J Koerner M McPhee A Wallman J (2008) An unexpected, stripe-faced flying fox in ice age rock art of Australia’s Kimberley. Antiquity 82(318). http://antiquity.ac.uk/projgall/pettigrew/index.html

[B74] Pledge N (1980) Giant echidnas in South Australia. South Australian Naturalist 55 (2): 27-30.

[B75] Prideaux GJ Gully GA Couzens AMC Ayliffe LK Jankowski NR Jacobs Z Roberts RG Hellstrom JC Gagan MK Hatcher LM (2010) Timing and dynamics of Late Pleistocene mammal extinctions in southwestern Australia. Proceedings of the National Academy of Sciences USA 107: 22157-22162. doi: 10.1073/pnas.1011073107 PMC3009796 21127262

[B76] Registrar General Perth (1902) Statistical register of the colony of Western Australia for 1900 and previous years. Perth, Australia: Wm. Alfred Watson, Government Printer.

[B77] Ripley D (1942) Trail of the money bird: 30,000 miles of adventure with a naturalist. Harper and Brothers, New York.

[B78] Roberts FHS (1953) The Australian species of *Aponomma* and *Amblyomma* (Ixodoidea). Australian Journal of Zoology 1: 111-161. doi: 10.1071/ZO9530111

[B79] Roberts FHS (1964) Further observations on the Australian species of *Aponomma* and *Amblyomma* with descriptions of the nymphs of *Amblyomma moreliae* (L. Koch) and *Amb. loculosum* Neumann (Acarina: Ixodidae). Australian Journal of Zoology 12: 288-313. doi: 10.1071/ZO9640288

[B80] Roberts FHS (1970) Australian ticks. CSIRO, Melbourne.

[B81] Roberts R Flannery TF Ayliffe LK Yoshida H Olley JM Prideaux GJ Laslett GM Baynes A Smith MA Jones R Smith BL (2001) New ages for the last Australian megafauna: continent-wide extinction about 46,000 years ago. Science 292: 1888-1892. doi: 10.1126/science.1060264 11397939

[B82] Robinson LE (1926) The genus *Amblyomma*. In: Nuttall GHF Robinson LE (Eds). Ticks: a monograph of the Ixodoidea. Part IV, Cambridge University Press, Cambridge: 1-302.

[B83] Rothschild M (1883) Dear Lord Rothschild: birds, butterflies, and history. Balaban, Glenside, Pennsylvania.

[B84] Rothschild W (1892) Descriptions of two new mammals from New Guinea. Proceedings of the Zoological Society of London 1892: 545-546.

[B85] Rothschild W (1903) Preliminary diagnosis of a new genus and species of kangaroo. Novitates Zoologicae10: 414.

[B86] Rothschild W (1904) Note on *Dendrodorcopsis woodwardi*. Novitates Zoologicae 10: 543.

[B87] Rothschild W (1905a) Notes on *Zaglossus* and description of a new subspecies of *Echidna hystrix*. Novitates Zoologicae 12: 305-306.

[B88] Rothschild W (1905b) Note on *Macropus rufus* Desm. with description of a new subspecies. Novitates Zoologicae 12: 508.

[B89] Rothschild W (1905c) Notes on two kangaroos from the “Northern Territory of South Australia” with descriptions of a new species. Novitates Zoologicae 12: 509-510.

[B90] Rothschild W (1907) Further notes on *Macropus magnus*. Novitates Zoologicae 14: 333.

[B91] Rothschild W (1913) Novitates Zoologicae 20: 188-191.

[B92] Rothschild W Dollman G (1936) The genus *Dendrolagus*. Transactions of the ZoologicalSociety of London 21: 477-551. doi: 10.1111/j.1096-3642.1936.tb00459.x

[B93] Rule SW Brook BW Haberle SG Turney CSM Kershaw AP Johnson CN (2012) The aftermath of megafaunal extinction: ecosystem transformation in Pleistocene Australia. Science 335: 1483-1486. doi: 10.1126/science.1214261 22442481

[B94] Schneider C Moritz CA (1999) Rainforest refugia and Australia’s Wet Tropics. Proceedings of the Royal Society Series B Biological Sciences 266: 191-196. doi: 10.1098/rspb.1999.0621

[B95] Shaw G (1792)*Myrmecophaga aculeata*. The Porcupine Ant-eater. Naturalists’ Miscellany 3(36). [unnumbered]

[B96] Shaw G (1799)*Platypus anatinus*. The Duck-billed Platypus. Naturalists’ Miscellany 10(118). [unnumbered]

[B97] Speck NH (1964) Introduction and summary description of the West Kimberley area. General Report on Lands of the West Kimberley Area, WA Melbourne, CSIRO, 9–23.

[B98] Speck NH Fitzgerald K Perry RA (1964) The pasture lands of the West Kimberley area. In: General Report on Lands of the West Kimberley Area, WA Melbourne, CSIRO, 175–191.

[B99] Start AN Burbidge AA McDowell MC McKenzie NL (2012) The status of non-volant mammals along a rainfall gradient in the south-west Kimberley, Western Australia. Australian Mammalogy 34: 36-48. doi: 10.1071/AM10026

[B100] Storr GM (1966) J. T. Tunney’s itinerary in Northern Australia 1901–1903. Emu 66: 59-65. doi: 10.1071/MU966059

[B101] Taylor FH (1913) Report of the entomologist. Report of the Australian Institute of Tropical Medicine 1911: 49-74.

[B102] Thomas O (1888) Catalogue of the Marsupialia and Monotremata in the collections of the British Museum (Natural History). British Museum (Natural History), London.

[B103] Thomas O (1901) On some kangaroos and bandicoots from Barrow Island, North-west Australia and the adjoining mainland. Novitates Zoologicae 8: 394-396.

[B104] Thomas O (1904a) On a collection of mammals made by Mr. J.T. Tunney in Arnhem Land, Northern Territory of South Australia. Novitates Zoologicae 11: 222-229.

[B105] Thomas O (1904b) On a new rock-wallaby from north-west Australia. Novitates Zoologicae 11: 365-366.

[B106] Thomas O (1904c) New species of *Pteropus, Mus*, and *Pogonomys* from the Australian region. Novitates Zoologicae 11: 597-600.

[B107] Thomas O (1907a) On the occurrence of *Acanthoglossus* in British New Guinea. Annals and Magazine of Natural History (series 7) 20: 293–294.

[B108] Thomas O (1907b) A new *Acanthoglossus* from the island of Salawatti. Annals and Magazine of Natural History (series 7) 20: 498–499.

[B109] Thomas O (1909) Two new mammals from N. Australia. Annals and Magazine of Natural History (series 8) 4: 197–198.

[B110] Thomas O Rothschild W (1922) On a new subspecies of *Zaglossus*, with remarks on other species of the genus. Annals and Magazine of Natural History (series 9) 10: 129–131.

[B111] Turney CSM Flannery TF Roberts RG Reid C Fifield LK Higham TFG Jacobs Z Kemp N Colhoun EA Kalin RM Ogle N (2008) Late-surviving megafauna in Tasmania, Australia, implicate human involvement in their extinction. Proceedings of the National Academy of Sciences USA 105: 12150-12153. 10.1073/pnas.0801360105 PMC2527880 18719103

[B112] Tyndale-Biscoe H (2005) Life of marsupials. CSIRO Publishing, Collingwood, Victoria.

[B113] Van Deusen HM George G (1969) Results of the Archbold Expeditions. No. 90. Notes on the echidnas (Mammalia: Tachyglossidae) of New Guinea. American Museum Novitates 2383: 1-23.

[B114] Van Dyck S Strahan R (Eds) (2005) The mammals of Australia. Third edition. Reed New Holland, Sydney.

[B115] Viallanes MH (1880) Observations on the salivary glands of the echidnas. Annals and Magazine of Natural History (series 5) 5: 83–84.

[B116] Walton DW (1988) Zoological catalogue of Australia, Volume 5. Mammalia. Bureau of Flora and Fauna, Canberra.

[B117] Walton DW Richardson BJ (1989) Fauna of Australia, Volume 1B, Mammalia. AGPS, Canberra.

[B118] Whittell M (1954) Notes on field-trips of J. T. Tunney. Emu 38: 322-326.

[B119] Williams SE (1997) Patterns of mammalian species richness in the Australian tropical rainforests: are extinctions during historical contractions of the rainforest the primary determinants of current regional patterns in biodiversity? Wildlife Research 24: 513–530.

[B120] Wood Jones F (1923) The mammals of South Australia. Part I. The monotremes and the carnivorous marsupials (the Ornithodelphia and the didactylous Didelphia). E.E. Rogers, Government Printer, Adelaide, Australia.

